# Polymorphisms of SLC19A1 80 G>A, MTHFR 677 C>T, and Tandem TS Repeats Influence Pharmacokinetics, Acute Liver Toxicity, and Vomiting in Children With Acute Lymphoblastic Leukemia Treated With High Doses of Methotrexate

**DOI:** 10.3389/fped.2020.00307

**Published:** 2020-06-16

**Authors:** Magdalena Cwiklinska, Malgorzata Czogala, Kinga Kwiecinska, Anna Madetko-Talowska, Malgorzata Szafarz, Katarzyna Pawinska, Aleksandra Wieczorek, Tomasz Klekawka, Magdalena Rej, Konrad Stepien, Przemyslaw Halubiec, Agnieszka Lazarczyk, Karol Miklusiak, Miroslaw Bik-Multanowski, Walentyna Balwierz, Szymon Skoczen

**Affiliations:** ^1^Department of Oncology and Hematology, University Children's Hospital, Kraków, Poland; ^2^Department of Pediatric Oncology and Hematology, Institute of Pediatrics, Jagiellonian University Medical College, Kraków, Poland; ^3^Department of Medical Genetics, Chair of Pediatrics, Institute of Pediatrics, Jagiellonian University Medical College, Kraków, Poland; ^4^Department of Pharmacokinetics and Physical Pharmacy, Faculty of Pharmacy, Jagiellonian University Medical College, Kraków, Poland; ^5^Student Scientific Group of Pediatric Oncology and Hematology, Jagiellonian University Medical College, Kraków, Poland

**Keywords:** acute lymphoblastic leukemia, children, genes, polymorphism, methotrexate, pharmacokinetics, toxicity

## Abstract

**Introduction:** High dose methotrexate (HD-Mtx) is highly effective and significantly improves overall acute lymphoblastic leukemia (ALL) patients survival. The pharmacodynamics of Mtx depends on the polymorphism of genes encoding proteins engaged in the folate metabolism pathway. The aim of the current study is to determine the relationship between variants of folate metabolism-related genes and the frequency of acute toxicities of HD-Mtx.

**Material and Methods:** A group of 133 patients aged 1.5–18.1 years (median: 6.3) was treated in accordance with the ALL-IC-2002 and ALL-IC-2009 protocols. The following polymorphisms were determined: 80 G>A *SLC19A1* (solute carrier family 19 member 1; rs1051266) with direct DNA sequencing, as well as 677 C>T *MTHFR* (methylenetetrahydrofolate reductase; rs1801133) and the tandem repeats of the *TS* (thymidylate synthase) with PCR technique. HD-Mtx organ toxicities were evaluated based on the laboratory tests results and the National Cancer Institute criteria.

**Results:** In patients with genotypes AA for *SLC19A1* and CC or CT for *MTHFR* Mtx steady state concentrations (C_ss_) and AUC_inf_ were distinctly higher. In patients with genotype 3R/3R for *TS* initial elimination rate constant was significantly higher (*P* = 0.003). Patients receiving Mtx at the dose of 5 g/m^2^ had lower clearance (4.35 vs. 8.92 L/h/m^2^) as compared to the ones receiving 2 g/m^2^ that indicates non-linear Mtx elimination at the higher dose. Liver impairment was the most frequently observed toxicity. The homozygous genotype was associated with a significantly higher incidence of hepatic toxicity for both the *SLC19A1* (*P* = 0.037) and *TS* (*P* = 0.002). Logistic regression analysis indicated an increased risk of vomiting for the 2R/3R genotype of the *TS* gene (OR 3.20, 95% CI 1.33–7.68, *P* = 0.009) and for vomiting and hepatic toxicity for the 3R/3R genotype (vomiting: OR 3.39, 95% CI 1.12–10.23, *P* = 0.031; liver toxicity: OR 2.28, 95% CI 1.05–4.95, *P* = 0.038). None of the acute toxicities differed between the analyzed dosing groups.

**Conclusions:** Determination of polymorphisms of *SLC19A1, MTHFR*, and *TS* genes might allow for a better prior selection of patients with higher risk of elevated Mtx levels. Our study is the first one to report the increased risk of hepatotoxicity and vomiting in patients with *TS* polymorphisms.

## Introduction

Acute lymphoblastic leukemia (ALL) is diagnosed in about 30% of children with neoplastic diseases and is the most common neoplasm in pediatric population (1). Methotrexate (Mtx) is one of the key chemotherapeutic agents used in a high doses (HD) treatment regimens of childhood ALL. Due to the observed severe toxicity, HD-Mtx, defined as Mtx doses ≥ 1 g/m^2^, requires a proper monitoring of drug elimination and an adequate leucovorin rescue administration. Nonetheless, in some of ALL patients toxic plasma concentrations of Mtx are observed, causing severe acute chemotherapy complications. The resulting modifications of treatment regimens might negatively affect overall patient survival (2, 3).

**Figure 1:**
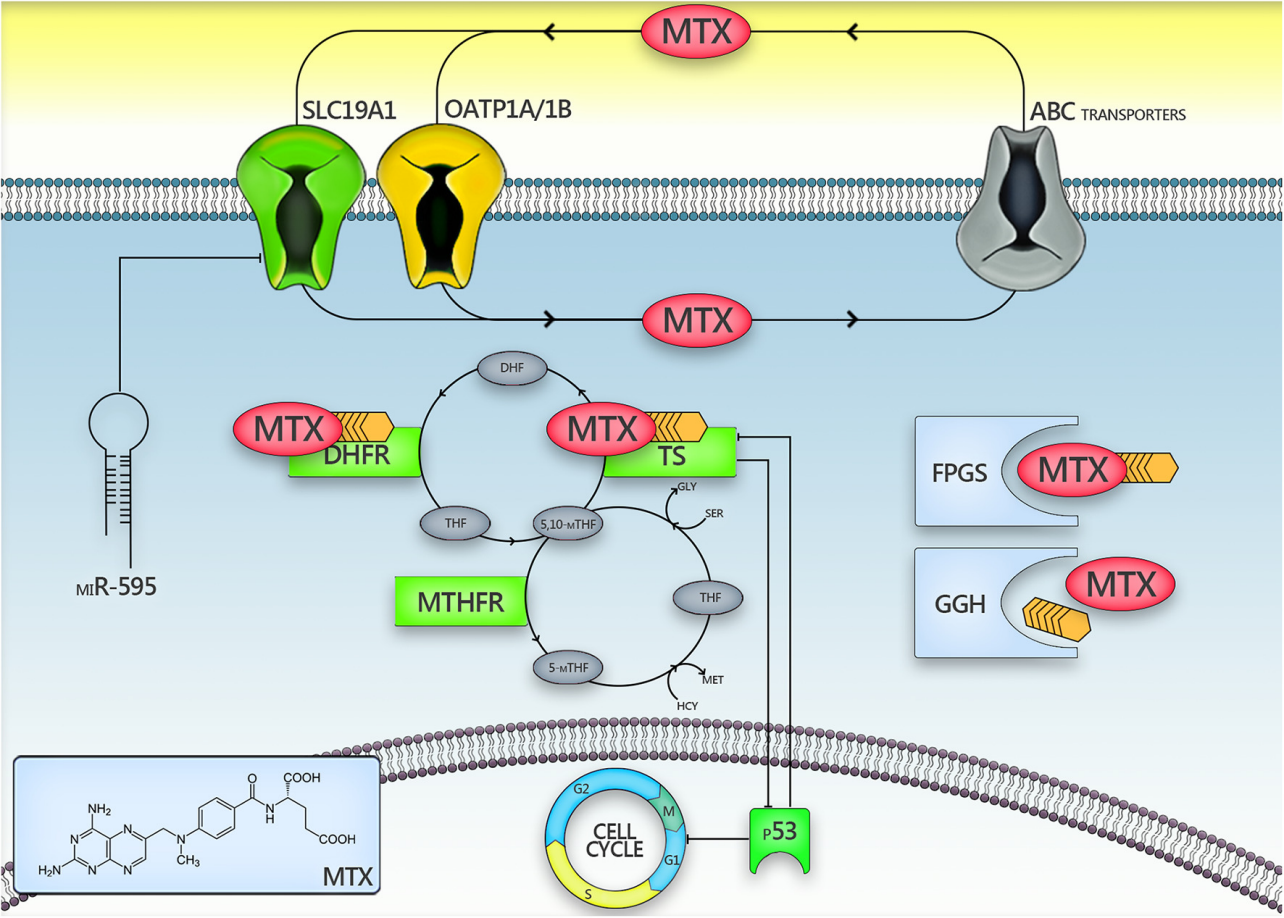
The presentation of intracellular methotrexate metabolism pathways. Factors that may be responsible for the inconsistency among studies assessing the role of *SLC19A1*/TS/MTHFR polymorphisms in methotrexate toxicity are marked with an asterisk (*).5,10-mTHF, 5,10-methylenetetrahydrofolate; ABC, ATP-binding cassette; DHFR, dihydrofolate reductase; FPGS, folylpolyglutamate synthase; GGH, γ-glutamyl transferase; miR-595, micro RNA 595; MTHFR, methylenetetrahydrofolate reductase; Mtx, methotrexate; OATP1A/1B, organic anion–transporting polypeptides 1A/1B; p53, p53 protein; *SLC19A1*, solute carrier family 19 member 1; TS, thymidylate synthase.

To date, well-known risk factors of toxicity after prolonged Mtx exposition include drug-drug interactions, insufficient prehydratation, older age, obesity or so called “third space fluid collections.” However, they do not explain all the changes observed in pharmacokinetics (PK) of Mtx in patients with childhood ALL. Numerous centers have performed comprehensive studies to explain the molecular basis of Mtx pharmacological activity and to identify genetic risk factors of its abnormal PK (3–6). As determined, Mtx enters the cell through the cell membrane by binding to the solute carrier family 19 member 1 (*SLC19A1*) (7–9). Inside the cell Mtx and its more active derivatives—polyglutamates block function of several enzymes of folate cycle, mainly dihydrofolate reductase (DHFR) responsible for production of active form of folate—tetrahydrofolate and thymidylate synthase (TS), involved in DNA synthesis (6, 10, 11). The final effect of Mtx pharmacological activity is blocking purine *de novo* synthesis and cells division. One of the main enzymes of the complex folate metabolism is methylenetetrahydrofolate reductase (MTHFR), that catalyzes the conversion of 5,10-methylenetetrahydrofolate to 5-methyltetrahydrofolate. Although Mtx does not directly inhibit MTHFR function, the activity of this enzyme is crucial for the body resources of tetrahydrofolate, that are necessary in DNA synthesis, as well as in methylation of DNA, lipids and proteins, including transformation of homocysteine into methionine. All aspects of Mtx disposition and mechanism of action that we attempt to consider are summarized in Figure 1.

As has been shown previously *SLC19A1, TS* and *MTHFR* genes polymorphisms are common in the European population (12, 13). Different variants of these proteins can influence cytotoxic effect of Mtx and contribute to acute side effects of HD-Mtx therapy (12, 13). Although published, until now, results regarding relationship between polymorphisms of *SLC19A1, TS*, and *MTHFR* genes, increased Mtx plasma concentrations and intensive toxicities caused by HD-Mtx indicated importance of genetic polymorphisms, they have been sometimes conflicting (6, 8, 14–16). There are also studies showing a clear relationship between the polymorphisms of above-mentioned genes involved in folate metabolism with worse therapeutic prognosis for children with ALL (14, 17).

The aims of the current study were to assess the prevalence of *SLC19A1* 80 G>A and *MTHFR* 677 C>T genes polymorphisms as well as *TS* gene tandem repeats in the group of children treated due to ALL and its influence on Mtx pharmacokinetics and incidence of acute toxicities caused by HD-Mtx.

## Materials and Methods

### Patients

The study group included 133 patients (Table 1), 1.5–18.1 years old (median: 6.3 years), treated in Department of Oncology and Hematology University Children's Hospital in Krakow, Poland, in accordance with ALL-IC-2002 (132 patients) and ALL-IC-2009 (1 patient) protocols. Both protocols for SR and IR risk pre-B ALL as well as T-ALL patients had the same frame. They were composed of induction (prednisone, vincristine, daunorubicin, L-asparaginase, cyclophosphamide, arabinoside cytosine, 6-mercaptopurine, and intrathecal methotrexate); consolidation (high dose methotrexate, 6-mercaptopurine); reinduction (dexamethasone, vincristine, doxorubicin, L-asparaginase, cyclophosphamide, arabinoside cytosine, 6-thioguanine and intrathecal methotrexate) and maintenance therapy (6-mercaptopurine, low dose methotrexate, and intrathecal methotrexate). The consolidation Mtx dose in ALL-IC-2002 was 2 g/m^2^ for all children with precursor-B, standard and intermediate risk ALL group. For T-ALL the dose was 5 g/m^2^. In ALL-IC-2009 the IR group and T-ALL were treated with Mtx at the dose of 5 g/m^2^, SR patients had the same dose of 2 g/m^2^. In both protocols the only additional drug given simultaneously was 6-mercaptopurine at the dose of 25 mg/m^2^. Together, 525 Mtx-chemotherapy cycles given in consolidation phase (protocol M) were studied. Two patients were given 1 cycle at the Mtx dose of 2 g/m^2^, and 3 cycles at the dose of 5 g/m^2^. One patient was given 3 cycles at the Mtx dose of 2 g/m^2^, and 1 cycle at the dose of 5 g/m^2^ (Table 1).

**Table 1:** Characteristic of patients according to Mtx dose.

**Mtx dose g/m^**2**^**	**Patients nb**.	**Chemotherapy cycles (%)**	**Age (median)**	**Gender (%)**	**BSA (%)**	**Type of ALL**	**Risk group**	**Cycles with delayed Mtx elimination (%)**
2	123	478 (91%)	1.7–16.2 (4.9)	64 girls (52) 59 boys (48)	75: N (61) 29: > 75p. (23.6) 19: <3 p. (15.4)	121–pre B 2–pre T	SR 58.5% IR 41.5%	69 (14.4)
5	13	47 (9%)	1.5–18.1 (7.3)	2 girl (15.4) 11 boys (84.6)	9: N (69.2) 3:>75 p. (23.1) 1: <3 p. (7.7)	1–pre B 12–pre T	IR 100%	28 (59.6)

*p., percentyl*.

### Genetic Analysis

Genetic analysis was performed in the laboratory with the international QC certificates (EMQN). DNA for molecular analyses was extracted with standard methods from blood mononuclears (0.5 ml of blood was collected from every patient; QIAamp DNA Blood Mini Kit was used, manufactured by QIAGEN). Assessment of 80 G>A *SLC19A1* polymorphism was performed with direct DNA sequencing (Sanger's method). In turn, 677C>T *MTHFR* polymorphism was analyzed with PCR-RFLP technique and *TS* tandem repeats were assessed based on the PCR with subsequent agarose gel electrophoresis (12). Based on genotyping results the patients were divided into 3 groups (“wild” genotype, heterozygotes, homozygotes). The sequences of primers that were used for genotyping were presented in the Supplementary Material.

### Pharmacokinetic Analysis

Pharmacokinetic parameters of Mtx were calculated based on the routinely measured concentrations after the HD-Mtx administration. The blood samples were taken at the end of 24 h infusion (steady state) and in the elimination phase at 36, 42, and 48 h from the beginning of the Mtx administration (i.e., 12, 18, and 24 h after the end of infusion). In the cases where the last concentration measured was above 0.4 μM (indication of prolonged Mtx elimination) subsequent samples were taken at the selected time points until the Mtx level decreased below 0.25 μM. Because of the potential for capacity-limited intracellular transport and renal clearance the elimination of Mtx is not accurately described by linear pharmacokinetic model. However, relatively simple two-compartment model appears to represent quite well the elimination phase. Therefore, the elimination constants (k_el_) for both initial α (up to 12 h after the end of infusion) and terminal β (from 12 to 24 h after the end of infusion) phases were calculated by log-linear regression of the drug concentration data in the appropriate phase. The area under the concentration vs. time curve extrapolated to infinity (AUC_inf_) was estimated using the log-linear trapezoidal rule and the total clearance (normalized per m^2^ of BSA) was calculated from dose/AUC_inf_. Mtx concentrations were analyzed with immunoenzymatic method on the VIVA-Vitalab analyzer, DADE-BEHRING, USA.

### Pharmacodynamic Analysis

The pharmacodynamics study was concentrated on the analysis of acute toxicity observed during the chemotherapy cycles at the Mtx doses of 2 and 5 g/m^2^. Every cycle, independent of HD-Mtx dose, was administered to a patient in good clinical condition, after exclusion of acute infection and with normal renal and liver functions. The routine blood tests panel included complete blood count, blood urea nitrogen, creatinine, total protein, albumin, bilirubin, alanine transaminase, aspartate transaminase, and electrolytes (all tests were measured in SI units). Tests were performed 1 day before HD-Mtx administration and 48 h after starting the infusion (24 h after the end of infusion). HD-Mtx toxicity was evaluated based on the analysis of laboratory tests results and clinical features according to the National Cancer Institute criteria (NCI 3.0 version). Liver (SGOT/SGPT, bilirubin), blood/bone marrow (WBC, PLT, Hgb, ANC) and gastrointestinal (vomiting, stomatitis) toxicities as well as concomitant infections were studied. Grades ≥ 2 according to NCI criteria were analyzed. Liver function was considered to be impaired if the following criteria (based on our own experience) were met: increase in transaminases level at least 1 grade and/or bilirubin grade ≥ 2 and/or decrease in protein level at least 13% comparing to the values observed before the actual cycle. Data concerning toxicity were collected prospectively at each cycle of chemotherapy, and were the basis for the subsequent therapeutic decisions, than all patients charts were reviewed.

### Statistical Analysis

Statistical analyses were performed with Statistica 12.0 (StatSoft, Statistica 12.0, Tulsa, Oklahoma, USA) software. Chi-square, Pearson chi-square and Fisher exact tests were used to identify relations between categorical variables. Comparison of numerical variables was performed using one-way ANOVA with *post-hoc* Tukey test or non-parametric Kruskal-Wallis test depending on the sample size. Allelic separation consistency within observed group of patients with expected allele distribution according to Hardy-Weinberg's rule was checked with use of the Chi-square test. Multiple logistic regression analysis was performed to identify risk factors of increased HD-Mtx therapy toxicities. Bonferroni correction for multiple comparisons was applied when assessing associations of toxicities and genetic variants, separately for each gene assessed. P-value of <0.05 was considered statistically significant.

## Results

The distribution of observed genotypes was consistent with the Hardy-Weinberg equilibrium (Table 2).

**Table 2:** The distribution of observed genotypes.

**Gene**	**Variant 1**	**Variant 2**	**Variant 3**	**Consistent with the Hardy-Weinberg**
80 G>A gene *SLC19A1*	GG−45 patients (33.8%)	AG−50 patients (37.6%)	AA−38 patients (28.6%)	(*P* = 0.054, χ^2^ = 5.84, df = 2)
677 C>T gene *MTHFR*	CC−66 patients (49.6%)	CT−54 patients (40.6%)	TT−13 patients (9.8%)	(*P* = 0.89, χ^2^ = 0.22, df = 2)
*TS* gene tandem repeats	2R/2R−29 patients (21.8%)	2R/3R−76 patients (57.1%)	3R/3R−28 patients (21.1%)	(*P* = 0.37, χ^2^ = 1.99, df = 2)

### Pharmacokinetic Results

**Figure 2:**
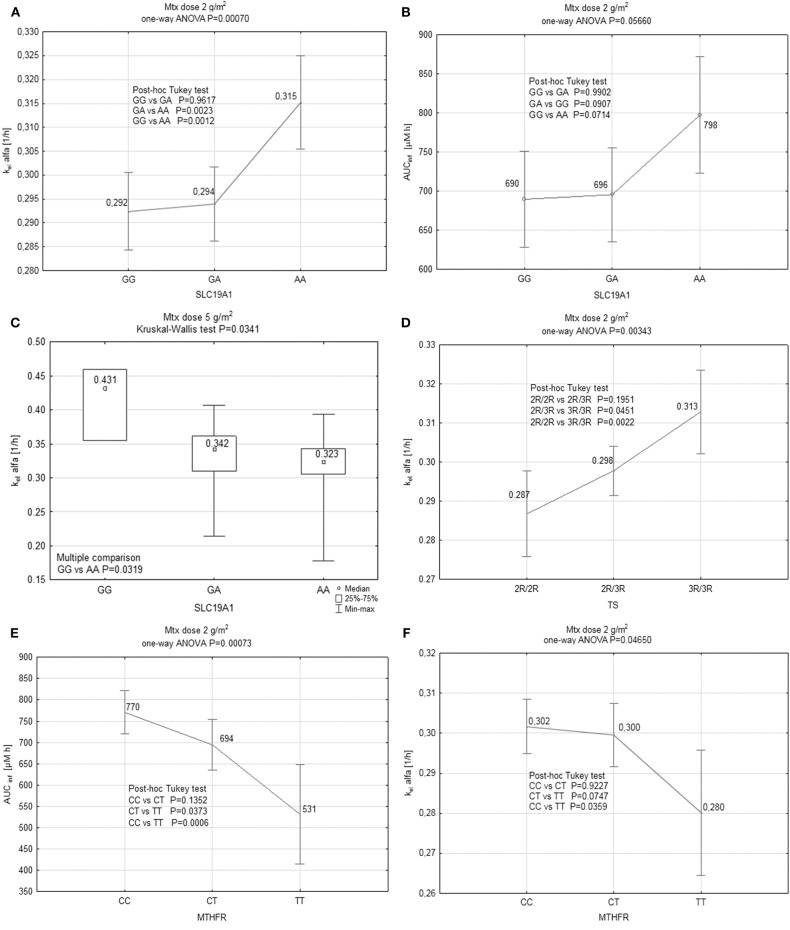
Results of analysis of influence of genetic polymorphisms on elimination of Mtx in both dosing groups: **(A)** Relationship between the initial elimination rate constant (k_el_ alfa) and SLC19A1 gene polymorphism in the patients receiving Mtx at the dose of 2 g/m^2^. Values are presented as mean and 95CI. **(B)** Relationship between the area under the concentration-time curve extrapolated to infinity (AUC_inf_) and SLC19A1 gene polymorphism in the patients receiving Mtx at the dose of 2 g/m^2^. Values are presented as mean and 95CI. **(C)** Relationship between the initial elimination rate constant (k_el_ alfa) and SLC19A1 gene polymorphism in the patients receiving Mtx at the dose of 5 g/m^2^. Values are presented as median and range. **(D)** Relationship between the initial elimination rate constant (k_el_ alfa) and TS gene polymorphism in the patients receiving Mtx at the dose of 2 g/m^2^. Values are presented as mean and 95CI. **(E)** Relationship between the area under the concentration-time curve extrapolated to infinity (AUC_inf_) and MTHFR gene polymorphism in the patients receiving Mtx at the dose of 2 g/m^2^ Values are presented as mean and 95CI. **(F)** Relationship between the initial elimination rate constant (k_el_ alfa) and MTHFR gene polymorphism in the patients receiving Mtx at the dose of 2 g/m^2^. Values are presented as mean and 95CI.

As expected, steady state concentrations of Mtx in patients treated with 5 g/m^2^ were significantly higher than in those receiving 2 g/m^2^ (137 vs. 38.5 μM). Moreover, the overall mean AUC_inf_ values were higher than proportionally expected (2,510 μM·h for 5 g/m^2^ vs. 717 μM·h for 2 g/m^2^) indicating lower total clearance in patients receiving Mtx at the dose of 5 g/m^2^ as compared to the ones receiving 2 g/m^2^ (4.35 vs. 8.92 L/h/m^2^, respectively). Furthermore, the percentage of patients with prolonged elimination, defined as the concentration > 0.4 μM at 48 h after the beginning of infusion, was much higher in the group receiving Mtx at the dose of 5 g/m^2^ (59.6 vs. 14.4%) (Table 1). These data might indicate that at the higher dose Mtx elimination process approaches saturation resulting in non-linearity of PK (Tables 3, 4). The possible variations in the Mtx levels resulting from non-linear elimination could influence the statistical analysis of the relationship between the genetic polymorphism and Mtx PK parameters. Moreover, due to limited number of observations, in the group receiving Mtx at the dose of 5 g/m^2^ the non-parametric statistical tests were used which are less powerful. Therefore, analysis of the influence of genetic polymorphism on PK of Mtx was mostly based on the parameters calculated after the dose of 2 g/m^2^. All the obtained results are presented in Tables 3, 4 as well as in Figure 2 (multiple comparison). Mean steady state concentrations of Mtx were significantly higher (42.9 vs. 36.9 or 37.3 μM) in homozygotes AA of 80 G>A gene *SLC19A1* polymorphism (*P* = 0.0467). Also homozygotes CC and heterozygotes 677 C>T of *MTHFR* gene had significantly higher (41.3 or 37.3 vs. 28.4 μM) mean Mtx steady state plasma concentrations in comparison to TT homozygotes (*P* = 0.0007). In the case of TS gene polymorphism slightly higher (42.9 vs. 35.9 or 37.9 μM) concentrations were observed for homozygotes 3R/3R for tandem repeats of the TS gene however the difference did not reach statistical significance. In the patients receiving Mtx at the dose of 2 g/m^2^ initial elimination rate constant and AUC_inf_ were significantly lower (*P* = 0.0465 and *P* = 0.00073, respectively) in homozygotes TT C>T of *MTHFR* gene, thus indicating higher clearance (12.5 vs. 8.42 L/h/m^2^ in e.g., homozygotes CC). Furthermore, the significant correlation has been found between initial elimination rate constant and polymorphism of *SLC19A1* and TS genes. Elimination rate constant was significantly higher in homozygotes 3R/3R for tandem repeats of the TS gene (*P* = 0.00343) and in homozygotes AA of 80 G>A gene *SLC19A1* (*P* = 0.0007), however in the latter case the AUC_inf_ was also higher (*P* = 0.05). On the contrary, for the dose of 5 g/m^2^ in homozygotes AA of 80 G>A gene *SLC19A1* initial elimination rate constant was significantly lower (GG vs. AA *P* = 0.0319) (Figure 2C). There was no significant influence of studied genetic polymorphism on the terminal elimination rate constant, although this is not a surprise since most of Mtx is eliminated during the α phase.

**Table 3:** Methotrexate (Mtx) steady-state concentrations and basic pharmacokinetic parameters, calculated after administration of Mtx at the dose of 2 g/m^2^, depending on the observed genotype.

	**Total (*n* = 478)**	**GG (*n* = 175)**	**GA (*n* = 180)**	**AA (*n* = 123)**	***P* value**
***SLC19A1*** **gene**					
Prolonged	69 (14.4%)	26 (14.9%)	31 (17.2%)	12 (9.8%)	NS
elimination					
C_SS_ [μM]	38.5 (36.5–40.5)	36.9 (33.6–40.1)	37.3 (34.4–40.2)	42.9 (38.3–47.6)	0.0467
k_el_ alfa [1/h]	0.30 (0.29–0.30)	0.29 (0.28–0.30)	0.29 (0.28–0.30)	0.31 (0.31–0.32)	0.0007
AUC_inf_ [μM·h]	717 (680–755)	690 (630–740)	696 (640–751)	797 (712–883)	0.0566
CL [L/h/m^2^]	8.92 (7.96–9.88)	9.51 (8.06–10.97)	9.25 (7.22–11.3)	7.40 (6.55–8.25)	NS
	**Total (*****n*** **=** **478)**	**CC (*****n*** **=** **246)**	**CT (*****n*** **=** **184)**	**TT (*****n*** **=** **48)**	***P*** **value**
***MTHFR*** **gene**					
Prolonged	69 (14.4%)	37 (15.0%)	25 (13.6%)	7 (14.6%)	NS
elimination					
C_SS_ [μM]	38.5 (36.5–40.5)	41.3 (38.3–44.3)	37.3 (34.3–40.3)	28.4 (24.4–32.9)	0.0007
k_el_ alfa [1/h]	0.30 (0.29–0.30)	0.30 (0.29–0.31)	0.30 (0.29–0.31)	0.28 (0.26–0.30)	0.0465
AUC_inf_ [μM·h]	717 (680–755)	770 (714–826)	694 (639–750)	531 (452–611)	0.0073
CL [L/h/m^2^]	8.92 (7.96–9.88)	8.42 (6.9–9.9)	8.66 (7.71–9.61)	12.50 (8.10–16.91)	0.0496
	**Total (*****n*** **=** **478)**	**2R2R (*****n*** **=** **100)**	**3R2R (*****n*** **=** **283)**	**3R3R (*****n*** **=** **95)**	***P*** **value**
***TS*** **gene**					
Prolonged	69 (14.4%)	12 (12.0%)	43 (15.2%)	14 (14.7%)	NS
elimination					
C_SS_ [μM]	38.5 (36.5–40.5)	35.9 (31.1–40.7)	37.9 (35.5–40.3)	42.9 (37.9–47.7)	NS
k_el_ alfa [1/h]	0.30 (0.29–0.30)	0.28 (0.27–0.30)	0.30 (0.29–0.30)	0.31 (0.30–0.32)	0.0034
AUC_inf_ [μM·h]	717 (680–755)	680 (589–771)	705 (661–751)	792 (702–881)	NS
CL [L/h/m^2^]	8.92 (7.96–9.88)	10.29 (7.88–12.70)	8.96 (7.62–10.3)	7.35 (6.31–8.38)	NS

*Values are given as mean and 95 CI. Comparisons were performed using one-way ANOVA. The number of patients with prolonged elimination (Mtx concentration measured at 48 h from the beginning of the infusion ≥ 0.4 μM) is given in both unrelative (N) and relative (%N) way. Pearson chi-square was used to identify relations between prolonged Mtx elimination and genotype. C_ss_, steady state concentration; k_el_ alfa, initial elimination rate constant; AUC_inf_, area under the concentration-time curve extrapolated to infinity; CL, clearance; NS, non significant*.

**Table 4:** Methotrexate (Mtx) steady-state concentrations and basic pharmacokinetic parameters, calculated after administration of Mtx at the dose of 5 g/m^2^, depending on the observed genotype.

	**Total (*n* = 47)**	**GG (*n* = 4)**	**GA (*n* = 20)**	**AA (*n* = 23)**	***P* value**
***SLC19A1*** **gene**					
Prolonged	28 (59.6%)	1 (25.0%)	14 (70.0%)	13 (56.5%)	NS
elimination					
C_SS_ [μM]	137 (24–230)	185 (139–230)	147 (42–200)	76 (24–200)	0.0015
k_el_ alfa [1/h]	0.33 (0.18–0.46)	0.43 (0.35–0.46)	0.34 (0.21–0.41)	0.32 (0.18–0.39)	0.0345
AUC_inf_ [μM·h]	2510 (446–5467)	3617 (2527–4216)	2718 (776–3645)	1392 (446–5467)	0.0019
CL [L/h/m^2^]	4.35 (0.80–24.6)	3.11 (2.61–4.35)	4.05 (3.02–14.17)	6.66 (0.8–24.6)	0.0295
	**Total (*****n*** **=** **47)**	**CC (*****n*** **=** **13)**	**CT (*****n*** **=** **30)**	**TT (*****n*** **=** **4)**	***P*** **value**
***MTHFR*** **gene**					
Prolonged	28 (59.6%)	11 (84.6%)	15 (50.0%)	2 (50.0%)	NS
elimination					
C_SS_ [μM]	137 (24–230)	140 (24–197)	138 (35–230)	109 (89–146)	NS
k_el_ alfa [1/h]	0.33 (0.18–0.46)	0.34 (0.27–0.37)	0.33 (0.18–0.46)	0.34 (0.21–0.36)	NS
AUC_inf_ [μM·h]	2510 (446–5467)	2551 (446–3603)	2519 (680–5467)	1985 (1626–2727)	NS
CL [L/h/m^2^]	4.35 (0.80–24.6)	4.31 (3.0–24.6)	4.28 (0.8–16)	5.55 (4.0–6.8)	NS
	**Total (*****n*** **=** **47)**	**2R2R (*****n*** **=** **16)**	**3R2R (*****n*** **=** **19)**	**3R3R (*****n*** **=** **12)**	***P*** **value**
***TS*** **gene**					
Prolonged	28 (59.6%)	7(43.8%)	13 (68.4%)	8 (66.7%)	NS
elimination					
C_SS_ [μM]	137 (24–230)	109 (24–200)	145 (44–230)	122 (35–195)	NS
k_el_ alfa [1/h]	0.33 (0.18–0.46)	0.34 (0.21–0.37)	0.33 (0.18–0.46)	0.32 (0.18–0.41)	NS
AUC_inf_ [μM·h]	2510 (446–5467)	1985 (446–3645)	2645 (861–5467)	2229 (680–3533)	NS
CL [L/h/m^2^]	4.35 (0.80–24.6)	5.55 (3.0–24.6)	3.89 (0.8–12.8)	5.0 (1.29–14.2)	NS

*Values are given as median and range. Comparisons were performed using Kruskal-Wallis test (small samples size). The number of patients with prolonged elimination (Mtx concentration measured at 48 h from the beginning of the infusion ≥ 0.4 μM) is given in both unrelative (N) and relative (%N) way. Pearson chi-square was used to identify relations between prolonged Mtx elimination and genotype. C_ss_, steady state concentration; k_el_ alfa, initial elimination rate constant; AUC_inf_, area under the concentration-time curve extrapolated to infinity; CL, clearance; NS, non significant*.

### Pharmacodynamic Results

In the case of homozygotes AA (80 G>A gene *SLC19A1* polymorphism), a statistical trend for higher incidence of transaminase elevation was observed (*P* = 0.037 without correction for multiple comparisons). Furthermore, similar trend was observed in the case of 3R/3R genotype of *TS* tandem repeats (*P* = 0.002 without correction for multiple comparisons or 0.01 with Bonferroni correction). No significant influence of all analyzed polymorphisms on the incidence of hematological toxicity, vomiting, gastrointestinal mucositis, and infections was observed (Table 5).

**Table 5:** Statistical significance (*Bonferroni correction for multiple comparisons) of particular acute toxicities depending on the three analyzed genes polymorphisms.

**Type of toxicity**	**AA *SLC19A1***,	**N (%)**	**TT *MTHFR***,	**N (%)**	**3R/3R**	***N* (%)**
	***P* value**		***P* value**		***TS***,	
					***P* value**	
Features of impaired liver function	0.037 (*NS)	110 (75.3)	0.609 (*NS)	38 (73.1)	0.002 (*0.01)	83 (76.9)
Hematological toxicity	0.657 (*NS)	17 (11.6)	0.248 (*NS)	5 (9.6)	0.453 (*NS)	18 (16.7)
Vomiting	0.056 (*NS)	19 (13.0)	0.682 (*NS)	7 (13.5)	0.102 (*NS)	15 (13.9)
Mucositis	0.590 (*NS)	10 (6.9)	0.341 (*NS)	2 (3.9)	0.207 (*NS)	14 (13.0)
Infections	0.056 (*NS)	12 (8.2)	0.424 (*NS)	6 (11.5)	0.560 (*NS)	7 (6.5)

*NS, non significant*.

Occurrences of particular acute toxicities induced by HD-Mtx were also analyzed with logistic regression models (Table 6). All commonly known risk factors of acute adverse reactions to HD-Mtx, such as: dose, prolonged drug exposure, age, as well as genotype were included. Heterozygous genotype 2R/3R of *TS* tandem repeats was associated with significant increase in stated intensive vomiting (OR adjusted to the wild-type genotype 3.20, 95% CI 1.33–7.68; *P* = 0.009). Similar relationship was also observed in the case of 3R/3R homozygotes (adjusted OR 3.39, 95% CI 1.12–10.23; *P* = 0.031). Additionally, as also demonstrated by logistic regression, 3R/3R polymorphism was associated with a higher risk of hepatotoxicity (adjusted OR 2.28, 95% CI 1.05–4.95; *P* = 0.038). No such relationships were observed for the other analyzed polymorphisms as well as for other acute toxicities.

**Table 6:** Logistic regression analysis of acute toxicities adjusted to prolonged exposure to methotrexate, drug dose, age, and genotype.

**Polymorphism**	**Genotype**	**Hepatotoxicity**	***P* value**	**Vomiting**	***P* value**
80 G>A *SLC19A1*	hom GG het GA hom AA	1.00 (–) 1.44 (0.81–2.55) 1.87 (0.96–3.63)	– NS 0.066	1.00 (–) 0.40 (0.16–1.02) 0.85 (0.35–2.03)	– 0.054 NS
2R>3R *TS*	hom 2R/2R het 2R/3R hom 3R/3R	1.00 (–) 1.46 (0.78–2.73) 2.28 (1.05–4.95)	– NS 0.038	1.00 (–) 3.20 (1.33–7.68) 3.39 (1.12–10.23)	– 0.009 0.031
677 C>T *MTHFR*	hom CC het CT hom TT	1.00 (–) 1.07 (0.63–1.81) 1.22 (0.65–2.29)	– NS NS	1.00 (–) 0.83 (0.43–1.63) 1.34 (0.32–5.59)	– NS NS

*Data are shown as odds ratios with 95% confidence intervals*.

*NS, non significant*.

### Impact of Particular Mtx Dosage (2 vs. 5 g/m^2^) on Toxicity

In this study, 525 chemotherapy cycles with 2 and 5 g/m^2^ Mtx doses (478 and 47 cycles, respectively) were analyzed. Impaired liver function, the most common acute toxicity, was observed with the same frequency in both groups. For the dosing of 2 and 5 g/m^2^ it was 68.4 and 66.7% of all patients, respectively (*P* = 0.816). Surprisingly, none of the acute toxicities differed between the analyzed groups (Table 7). Only an insignificant relationship was observed toward a higher incidence of hematological toxicity in the group treated with higher doses of Mtx (12.3 vs. 22.9%, *P* = 0.124) (Table 7). In all patients adequate antitoxic therapy according to the requirements of protocols prevented life-threatening complications.

**Table 7:** The incidence of chemotherapy toxicities depending on the methotrexate doses.

**Toxicity**	**2 g/m^**2**^**	**5 g/m^**2**^**	***P***
	***N* (%)**	***N* (%)**	**value**
Impaired liver function	327 (68.4)	32 (66.7)	NS
Vomiting	52 (10.9)	9 (18.8)	NS
Stomatitis/skin inflammation	42 (8.8)	4 (8.3)	NS
Infections	39 (8.2)	4 (8.3)	NS
Hematological toxicity	59 (12.3)	11 (22.9)	NS

*NS, non significant*.

## Discussion

We showed in our study that the genetic polymorphisms of the *SLC19A1, MTHFR*, and *TS* genes can influence pharmacokinetics of Mtx. Importantly, we have observed, for the first time in the literature, the increased risk of hepatotoxicity (significant even with Bonferroni correction) and vomiting in patients with particular *TS* polymorphism and for the second time the increased risk of hepatotoxicity in the *SLC19A1* homozygous genotype. Surprisingly, the Mtx dose did not affect the incidence of individual toxicities, which may indicate a congenital predisposition to their development in individual ALL patients.

### *SLC19A1* 80 G>A Polymorphism and Its Influence on Hepatotoxicity

*SLC19A1* 80 G>A is a common single nucleotide polymorphism among genes responsible for Mtx transport into a cell (6, 14, 17–20). Our results indicate a relationship between the AA genotype of the *SLC19A1* 80 G>A polymorphism and significantly elevated steady state Mtx concentrations after HD-Mtx infusions (e.g., 42.9 vs. 36.9 μM) (Table 3). Since this particular mutation is responsible for the lower affinity of the transporter protein to Mtx, it is expected that in these patients the higher amount of drug stays in the central circulation. Although patients, with this genotype, receiving the lower dose of Mtx had higher initial elimination rate constant (e.g., 0.315 vs. 0.292 1/h for AA vs. GG; *P* = 0.0012) (Figure 2A), the total clearance was lower (higher AUC_inf;_ e.g., 798 vs. 690 μM·h for AA vs. GG) (Figure 2B). On the contrary patients with AA genotype of the *SLC19A1* receiving 5 g/m^2^ of Mtx had significantly lower initial elimination rate constant (e.g., 0.323 vs. 0.431 1/h for AA vs. GG; *P* = 0.0319) (Figure 2C). However, these changes are rather consequences of the saturation of Mtx elimination processes. Higher Mtx exposure in AA homozygotes might result in the impaired liver function, defined by us as increase in transaminases level at least 1 grade and/or bilirubin grade ≥ 2 and/or decrease protein level, that was observed significantly more frequently (not significant with Bonferroni correction) in these patients. Until now, only one study showed a correlation between the 80 AA variant and significant liver function impairment caused by Mtx. Moreover, it was referred only to the group of patients with an additional, specific variant of *GSTM1* gene (15). The mechanism explaining increased liver toxicity, despite the presence of a variant of reduced folate carrier (RFC) protein with lower ability to transport Mtx (also into hepatocytes), may involve the participation of other transporters, with higher expression in liver tissues. The explanation could be the activity of OATP1A/1B which determine transport of drugs (including Mtx) into hepatocytes (21), alteration of the polyglutamylation pattern (22) or the intracellular level of miR-595 acting as a phenotypic regulator of Mtx sensitivity in cells by targeting *SLC19A1* (23) (Figure 1).

The association between *SLC19A1* polymorphism and Mtx levels, similar to the one observed in our study, was also shown by Laverdiére et al. (9). Since then, several researchers have observed the impact of this polymorphism on incidence of specific adverse reactions. Kotnik et al. showed an association of AA genotype with leukopenia, while Gregers et al. with significant percentage of serious myelotoxicities (6, 24). In the study conducted by Salazar et al. the wild GG genotype was associated with thrombocytopenia and mucositis of grade at least 2 according to WHO (14). However, the opposite results were presented by some other researchers who did not find the impact of *SLC19A1* polymorphism on acute toxicities of HD-Mtx therapy, although liver toxicity was not investigated (3, 25–28).

It should be emphasized that the number of patients in our study almost 3-fold exceeds the total number of HD-Mtx treated patients reported in previous studies of Mtx hepatotoxicity. Thus, our results showing possible increased risk of HD-Mtx induced hepatotoxicity in patients with the *SLC19A1* 80 AA variant seem to be better substantiated. It should also be emphasized that previous studies were based on assessment of various ethnic groups and of patient cohorts of variable size. The polymorphisms of numerous genes involved in Mtx disposition might differ among various populations thus possibly determining the toxic effects of treatment (29).

### The Role of *TS* Repeats and Their Impact on Hepatotoxicity and Vomiting

The results of our study suggest that determination of the *TS* gene polymorphism in pediatric population may have significant clinical implications in predicting liver impairment associated with HD-Mtx (significant even with Bonferroni correction).

Mtx is an uncompetitive, irreversible inhibitor of TS and, from a pharmacokinetic point of view, this enzyme should have a relatively low contribution to Mtx elimination (30). However, slightly higher (without statistical significance) steady state Mtx concentrations were seen in homozygotes 3R/3R for tandem repeats of the *TS* gene (e.g., 42.9 vs. 35.9 μM for 3R/3R vs. 2R/2R) (Table 3). Since simultaneous increase in the initial elimination rate has been also observed (e.g., 0.313 vs. 0.287 1/h for 3R/3R vs. 2R/2R; *P* = 0.0022) (Figure 2D) the decrease in total clearance in the patients with 3R/3R variant for tandem repeats of the *TS* gene, although visible (7.35 vs. 10.29 L/h/m^2^), did not reach statistical significance (Table 3). The vast majority of Mtx is eliminated by renal route and the higher amount of proteins able to irreversibly bind Mtx might impair the elimination process, but this hypothesis needs further investigation. However, it should also be stressed that TS plays additional (not directly related to folate metabolism) role in cell homeostasis control through associations with numerous cell cycle proteins, in particular with p53. The mutual regulation of TS and p53 is based on negative feedback loop. In 3R/3R cells with the higher TS expression the p53 level will be lower (31), what decreases ability of intensively dividing cells (e.g., hepatocytes and gastrointestinal epithelium cells) to block the cell cycle during exposure to the severely damaging factors acting during the S phase (such as Mtx). It may explain the increased incidence of mucosal and liver damage observed in our study (32). The published results regarding the influence of TS gene polymorphism on the incidence of different toxicities are not consistent. Ongaro et al. showed a significant increase in the risk of anemia in adult patients with 3R/3R genotype, while other researchers reported increased toxicity in the central nervous system (33–37). Opposite results were presented by Kotnik et al. and Erculj et al. showing that 3R/3R genotype was associated with a reduced risk of mucositis, leukopenia and thrombocytopenia (6, 38). Demonstrated by us relationship between 3R/3R genotype and increased hepatotoxicity, as we mentioned earlier, has not been reported previously.

### The Role of *MTHFR* 677 C>T Polymorphism in Toxicity of HD-Mtx

In our study, patients homozygotes CC and heterozygous for the common 677C>T polymorphism of the *MTHFR* gene achieved significantly higher steady state Mtx plasma concentrations (41.3 or 37.3 vs. 28.4 μM; *P* = 0.0007) (Table 3). Elevated levels of Mtx in the carriers of CT variant of the *MTHFR* gene are consistent with several previous observations (16, 39, 40). However, we did not show significant differences in the frequency of acute side-effects of HD-Mtx in carriers of this polymorphism. Such results observed in our study could be partially explained by the fact that despite the higher steady state concentrations and higher AUC_inf_ values (694 vs. 531 μM·h for CT vs. TT; *P* = 0.0373) (Figure 2E) the carriers of this gene mutation had also slightly higher, although not significantly, initial elimination rate constant (0.3 vs. 0.28 1/h) (Figure 2F) indicating faster decrease of Mtx concentration immediately after the end of infusion. Lack of relationships between toxicity and *MTHFR* gene polymorphism have already been noticed by Seidemann et al. (41) and Shimasaki et al. (16), except for mucosal toxicity directly caused by higher drug plasma concentrations. However, other authors found higher frequency of mucosal toxicity or increased hematological toxicity associated with *MTHFR* gene polymorphism (29, 42, 43). El-Khodary et al. described increased hepatic and myeloid toxicity in *MTHFR* 677TT homozygotes (44). The presence of the 677T allele was also associated with a higher risk of thrombocytopenia (45), and an overall increase in toxicity, if in combination with the 1298AC variant (46). Considering the conflicting reports presented in the literature it is highly possible that, in the case of 677C>T *MTHFR* gene polymorphism some other concomitant factors are likely to affect toxicity of HD-Mtx treatment.

### Limitations of the Study

Our study has some limitations. First, the tested polymorphisms seem to be important for the Mtx disposition, but the influence of other genetic and biochemical factors should be also considered. The influence of combinations of specific alleles of several genes, including possible synergistic or antagonistic effects are possible. Second, more advanced genetic methods, such as whole exome sequencing, could provide more information on other potential polymorphisms associated with Mtx toxicity (47, 48). Third, our study group was homogenous as far as ethnicity is concerned, so this makes the discussion focused solely on the central European population.

## Conclusions

The genetic polymorphisms has an unquestionable effect on pharmacokinetics and toxicity of Mtx. Determination of polymorphisms of *SLC19A1, MTHFR*, and *TS* genes allows for a better selection of patients with higher risk of elevated Mtx levels. According to our knowledge, our study is the first one to report the increased risk of hepatotoxicity and vomiting in patients with particular *TS* polymorphisms. In addition, we were able to confirm the previous data showing that the increased risk of hepatotoxicity has been associated with the *SLC19A1* homozygous genotype. Surprisingly, the administered Mtx dose did not affect the incidence of individual toxicities. Further research, considering also polymorphisms of other folate metabolism pathways and some mutual gene associations, including meta-analyzes of the previous studies, is necessary for the final determination of the role of individual polymorphisms in the pharmacokinetics, pharmacological activity, and toxicity of Mtx. Such studies could lead to the pharmacogenetically improved, individualized dosing of Mtx, that in turn could compensate for its interindividual PK variations.

## Data Availability Statement

The datasets generated for this study are available on request to the corresponding author.

## Ethics Statement

The studies involving human participants were reviewed and approved by local Permanent Ethical Committee for Clinical Studies (KBET/96/B/2008). Written informed consent to participate in this study was provided by the participants' legal guardian/next of kin.

## Author Contributions

MCw, MCz, KK, AM-T, MB-M, WB, and SS contributed to the study concept and design. MCw, MCz, KK, AM-T, KP, AW, TK, and MR performed diagnostic tests and collected relevant clinical data. MCw, MCz, AM-T, MS, KS, PH, AL, and KM conducted statistical analysis. MCw, MCz, MS, KS, PH, AL, and KM wrote sections of the manuscript. MB-M, WB, and SS critically revised the article. All authors were responsible for the integrity and accuracy of the data and approved the submitted version.

## Conflict of Interest

The authors declare that the research was conducted in the absence of any commercial or financial relationships that could be construed as a potential conflict of interest.
