# Supplementary material for: Polymorphisms of SLC19A1 80 G>A, MTHFR 677 C>T, and Tandem TS Repeats Influence Pharmacokinetics, Acute Liver Toxicity, and Vomiting in Children With Acute Lymphoblastic Leukemia Treated With High Doses of Methotrexate
Source: Front Pediatr. 2020 Jun 16;8:307. doi: 10.3389/fped.2020.00307 (PMC7308427; doi:10.3389/fped.2020.00307)
Supplement: Supplementary file 1 [file Data_Sheet_1.DOCX]

Supplementary material

Sequences of primers that were used for genotyping

MTHFR gene

F: TGAAGGAGAAGGTGTCTGCGGGA

R: AGGACGGTGCGGTGAGAGTG

TS gene

F: GTGGCTCCTGCGTTTCCCCC

R: CCAAGCTTGGCTCCGAGCCGGCCA

SLC19A1 gene

F: CTTCCAAGGTGCCCTGACTC

R: CACCCACCCACAGGCG
